# Comparative Assessment of Matrix-assisted Laser Desorption Ionization-time of Flight Mass Spectrometry (MALDI-TOF-MS) and Conventional Methods in the Identification of Clinically Relevant Yeasts

**DOI:** 10.7759/cureus.15607

**Published:** 2021-06-11

**Authors:** Suneeta Meena, Aroop Mohanty, Neelam Kaistha, U Sasirekha, Jitendra Meena

**Affiliations:** 1 Lab Medicine, All India Institute of Medical Sciences New Delhi, New Delhi, IND; 2 Microbiology, All India Institute of Medical Sciences Gorakhpur, Gorakhpur, IND; 3 Microbiology, All India Institute of Medical Sciences Rishikesh, Rishikesh, IND; 4 Preventive Oncology, National Cancer Institute, All India Institute of Medical Sciences Jhajjar, Haryana, IND

**Keywords:** yeast, maldi-tof-ms, candida, identification, cma

## Abstract

Background: Candida species are generally identified by conventional methods such as germ tube or morphological appearance on cornmeal agar (CMA), biochemical methods using API kits, and molecular biological techniques. Matrix-assisted laser desorption ionization-time of flight mass spectrometry (MALDI-TOF-MS) has revolutionized the identification of fungi reducing the turnaround time of days to minutes.

Purpose: To compare the performance of MALDI-TOF MS and conventional methods in the identification of clinically relevant yeasts.

Materials and methods: In this study, Candida identifications on CMA are compared with the results obtained on MALDI-TOF MS (Bruker Daltonics, Bremen, Germany). Discrepant results were confirmed by sequencing internal transcribed spacer (ITS) regions of rDNA.

Results: A total of 114 clinical Candida species isolated from blood cultures were isolated and identified with conventional methods as well as with the MALDI-TOF-MS system. The agreement between the two test results were analyzed using Inter-rater reliability analysis (Cohen's Kappa) in SPSS Software Version 24 (IBM Corp., Armonk, NY). Overall, there was substantial agreement (Cohen's kappa=0.763) between the two methods. A value between 0.61 and 0.80 is classified under substantial. The most frequently isolated bloodstream Candida species included *Candida albicans, C. tropicalis*, *C. parapsilosis, C.lusitaniae, C. glabrata* which were accurately identified by MALDI-TOF-MS. When compared with conventional identification methods, MALDI-TOF-MS results are more reliable and rapid for Candida identification.

## Introduction

Identifications of yeasts in the mycology laboratory are carried out by phenotypic methods like germ tube tests and Dalmau plate culture. All these methods can be accurate in the hands of an experienced mycologist, but it takes a minimum of 48 hours for the identification process. Molecular techniques, such as real-time PCR, gene sequencing give accurate fungal identification, but they come at a very high cost and require highly-trained technicians. A faster identification would not only decrease the turnaround time of the laboratory but would also enhance the throughput. In addition, quick and dependable identification of species is of utmost importance in order to start the correct antifungal treatment due to species-specific susceptibility patterns [1].

One of the most common reasons for the increasing mortality due to invasive fungal infections is the large turnaround time resulting in delay in the initiation of therapy. This high mortality rate might be substantially reduced by early interference with the antifungal drugs, which might lead to effective management of the high-risk patients [2]. Matrix-assisted laser desorption ionization-time of flight mass spectrometry (MALDI-TOF-MS) has revolutionized the identification of fungi giving results in minutes; therefore, it is becoming essential equipment in clinical mycology laboratories [3]. This modern technology compares the unique mass spectral fingerprints generated from the ionization of the different proteins with the gigantic library of mass spectra already present within the system. As these spectral fingerprints are distinctive for each individual microorganism, accurate microbial identification both at the genus and species level is possible using bioinformatics pattern profiling [4]. Here, in our study, we evaluated the performance of the MALDI-TOF-MS (Bruker Daltonics, Bremen, Germany) with that of the conventional methods for the identification of Candida strains.

## Materials and methods

This is a cross-sectional study conducted by the Mycology Laboratory, Department of Microbiology, AIIMS Rishikesh which is a tertiary care level institute in Uttarakhand state. We included all the Candida spp. isolated from positive blood cultures reported in the year 2019. These blood culture bottles received from the wards were incubated in the BacT ALERT 3D Microbial Identification system (Biomerieux, Boston, USA) automated blood culture system. Gram staining was done from all the bottles giving a positive signal to look for budding yeast cells. Sabouraud’s dextrose agar and 5% sheep blood agar were used for culturing these positive isolates. They were incubated at 37 °C for 48 hours. Candida species were isolated and identified with conventional methods such as germ tube test, HiCHROM agar (Himedia, Mumbai, India), and Dalmau plate culture. All isolates were inoculated onto cornmeal agar (CMA) agar (Himedia) and incubated under necessary conditions. Finally, all isolates were identified under a microscope (400× magnification) according to their chlamydospore, blastospore, and/or hypha formation.

MALDI-TOF MS analyses

A colony of yeast was picked using an inoculation loop and was dissolved in formic acid. One microliter of this suspension was then spotted onto the MALDI plate, allowed to dry, and then overlaid with the matrix solution consisting of 40 g of alpha-cyano-4-hydroxycinnamic acid (CHCA; Sigma-Aldrich, Buchs, Switzerland) in 33% ethanol, 33% deionized water, 33% acetonitrile (ACN; Sigma-Aldrich), and 3% trifluoroacetic acid (TFA) [5]. The matrix was then dried in room air. The MALDI plate was loaded onto the equipment. Each MALDI plate was externally calibrated using a reference strain of *Escherichia coli* DH5α (Invitrogen, Carlsbad, USA) that was also simultaneously spotted onto the plates each time.

Data analyses

Data were entered in MS Excel Software, Microsoft, Inc. (Redmond, WA, USA), and test reports using two methods (conventional vs. MALDI-TOF MS) were compared. Statistical agreement between two test results were analysed using inter-rater reliability analysis (Cohen's Kappa) in SPSS Software Version 24, IBM, Inc. P-value less than 0.05 was considered to be significant statistically.

## Results

Over 114 clinical candida spp. obtained from positive blood cultures within a year were isolated and identified using both the conventional and automated methods. Overall, there was a substantial agreement (kappa=0.763) between the two methods (Table 1). All the common Candida spp. (*C. albicans*, *C. tropicalis,* and *C. krusei* strains) were correctly recognized with conventional methods. Hi-CHROM Candida medium yielded better results after 48 hours of incubation because the colour of the colonies was more distinguishing. Both *C. parapsilosis* and C. glabrata strains were indistinguishable as the colour could not be differentiated. Besides, there were limitations for the identification of strains other than the common Candida spp. Isolates were also identified based on the morphology of Dalmau plate culture.

**Table 1:** Comparison of conventional and MALDI-TOF MS for identification of Candida spp

S.no.	Pathogenic yeast	Total isolates	CMA	MALDI-TOF	Cohen’s kappa	95% CI	Degree of agreement
1	C. albicans	41	41	41	0.981	(0.975, 0.987)	Almost perfect
2	C. tropicalis	32	31	31	1.000	(0.994, 1.006)	Almost perfect
3	C. parapsilosis	13	11	13	0.865	(0.859, 0.871)	Almost perfect
4	C. krusei	4	4	1	0.491	(0.485, 0.971)	Moderate
5	C. glabrata	4	4	4	0.791	(0.785, 0.797)	Substantial
6	C. lusitanae	5	3	3	0.653	(0.647, 0.659)	Substantial
7	C. auris	1	0	1	−0.004	(−0.010, 0.001)	Poor
8	Wickerhamomyces anomalus	5	0	3	−0.022	(−0.028, −0.017)	Poor
9	C. gulliermondii	1	1	1	1.000	(0.994, 1.006)	Almost perfect
10	Trichosporon asaahii	2	0	2	−0.004	(−0.010, 0.001)	Poor
11	T. duhanse	1	0	1	−0.004	(−0.010, 0.001)	Poor
12	T. ovoides	1	0	1	−0.004	(−0.010, 0.001)	Poor
13	C. neoformans var grubii	1	0	1	−0.004	(−0.010, 0.001)	Poor
14	C. neoformans var gattii	1	0	1	−0.004	(−0.010, 0.001)	Poor
15	Kodamaea ohmeri	1	0	1	−0.004	(−0.010, 0.001)	Poor
16	C. dubliensis	1	1	1	1.000	(0.994, 1.006)	Almost perfect
	Total	114	96/114	106/114	0.763	(0.759, 0.765)	Substantial

## Discussion

*C. albicans *and *C. tropicalis* were accurately identified to the species level by both methods. However, there were some discrepancies in identification by two methods. *C. krusei* in two patients were identified as *Wickerhamomyces* *anomalous*. *Trichosporon spp*. could only be identified up to the genus level by conventional methods. Whereas, MALDI-TOF MS could identify till species level picking up rare species like *T. dohaense.* One isolate identified as *T. ovoides* by MALDI-TOF MS was later confirmed as *C. krusei* by internal transcribed spacer (ITS) sequencing.

Identification by MALDI-TOF MS only took 15 min in our study. Previous studies have also concluded that the use of automated instruments like MALDI-TOF-MS for fungal identification has reduced the turnaround time, hospital stays, and costs as compared to conventional methods [5,6]. On average, a minimum of 24-48 hours is required for the identification of yeast-like fungi with phenotypic methods. This time may even go up to 72 hours for germ tube negative candida spp. Besides, rapid recognition of the organism helps in not only the optimal management of patients but also antifungal stewardship with optimal utilization of drugs [1].

In this study, the majority of the isolates were identified correctly with both the conventional and MALDI-TOF MS systems. In a recently published study, it was reported that the accurate identification ratio of MALDI-TOF-MS and conventional methods was 98.3% and 96.5 %, respectively, when rDNA sequence analysis was the reference [7,8]. Over 86% of our isolates were the commonly encountered Candida spp. causing bloodstream infections. These included *C. albicans*, *C.* *tropicalis*, *C. parapsilosis,* *C. lusitaniae*, *C. glabrata,* and *C. kruse*i. As a result, MALDI TOF-MS successfully identified all tested Candida and proved to be a rapid, reliable, and cost-effective method for identification, especially of the most common Candida strains [1]. It could also identify *Cryptococcus neoformans var. grubii *with a score of more than 2.0 thus helping in the species level classification.

However, four of our isolates showed discrepancy and were subjected to ITS region sequencing as a reference method. Two isolates of *C. krusei* were misidentified as *Wickerhamomyces anomalous *by MALDI-TOF MS. Similarly, another strain of *C. krusei* was misidentified as *T. ovoides *by MALDI-TOF MS. The possible result for discordant results from MALDI-TOF MS could be the use of a simple extraction method. Several studies have reported both increases in sensitivity and score value using a multipart extraction protocol [9,10]. In a recent study on *C. krusei*, it was demonstrated that early fluconazole therapy did not affect the mortality rate. Thus, the correct Identification of *C. krusei *could find a survival benefit when early, appropriate antifungal therapy is administered [11].

The MALDI-TOF-MS showed excellent performance in identifying rare *Trichosporon *species like *T. dohaense*, wherein conventional methods can only identify this organism to the genus level. Biochemical profiling methods are also inadequate for the steadfast Identification of Trichosporon species [12]. Identification of fungal pathogens to the species level is essential for the prescription of the appropriate antifungal therapy.

MALDI-TOF-MS is now practically the gold standard for the identification of *Candida auris*. Identification of this fungus is not only important but also urgent given the intensifying pattern similar to that of a bacterium. So, a rapid identification is the need of the times for prompt management of the patient.

Besides, another important finding of the study was that MALDI-TOF MS plays a vital role in the identification of yeast, which shows only blastoconidia on Dalmau plate culture. *C. glabrata*, *C. auris*, *Kodamaeaa ohmeri*, *C. famata*, Cryptococcus are some yeasts that do not show any characteristic chlamydospore, hyphae, or pseudohyphae formation. It helped us in identifying rare fungi in really sick and immunocompromised patients which would be very difficult to be recognized by simple methods and thus helped us in saving a lot of lives.

## Conclusions

In conclusion, our study indicates a substantial agreement between both the conventional and the automated methods used to identify the isolates of Candida species identified from the blood cultures. The MALDI-TOF-MS could very easily identify the common candida species whereas the other rare species were not identified conventionally. The MALDI-TOF-MS has reduced significantly the time taken to identify the fungal pathogens. Besides this, it has also helped in identifying the rare and cryptic species which never would have been identified by the earlier methods. Early and rapid identification helps in giving us an ample amount of time to perform the antifungal susceptibility testing and initiation of the correct antifungal therapy at the earliest. With the advent of many fungal infections in this COVID-19 pandemic, the use of this instrument will help us in the complete and species-level identification thus helping us to detect new variants.
